# USDA’s revised biotechnology regulation’s contribution to increasing agricultural sustainability and responding to climate change

**DOI:** 10.3389/fpls.2022.1055529

**Published:** 2022-11-23

**Authors:** Neil E. Hoffman

**Affiliations:** Biotechnology Regulatory Services, Animal and Plant Health Inspection Service, United States Department of Agriculture, Riverdale, MD, United States

**Keywords:** genome editing, regulatory policy, genetic engineering, plant biotechnology, environmental protection

## Abstract

Biotechnology can provide a valuable tool to meet UN Sustainable Development Goals and U.S. initiatives to find climate solutions and improve agricultural sustainability. The literature contains hundreds of examples of crops that may serve this purpose, yet most remain un-launched due to high regulatory barriers. Recently the USDA revised its biotechnology regulations to make them more risk-proportionate, science-based, and streamlined. Here, we review some of the promising leads that may enable agriculture to contribute to UN sustainability goals. We further describe and discuss how the revised biotechnology regulation would hypothetically apply to these cases.

## Introduction

The UN Sustainable Development Goals (SDG) are critically important for humanity and the planet (United Nations, 2015). Agricultural innovation can positively contribute to many of these goals such as ending hunger, promoting sustainable agriculture or clean energy, to name a few (Secretary-General, 2019). Through Executive Order #14008 (Executive Office of the President, 2021), the Biden Administration prioritized building a modern sustainable infrastructure and an equitable clean energy future demonstrating a commitment to many of these same goals.

Biotechnology has contributed to agricultural sustainability through traits that reduce over the top insecticide application. (Brookes and Barfoot, 2017). Regulatory barriers have limited both diverse trait development and developers who use biotechnology (Bradford et al., 2005; Hoffman, 2021). In May 2020, the U.S. Department of Agriculture (USDA) issued revised biotechnology regulations that offer a more risk-proportionate approach and are expected to spur innovation while ensuring products are safe for agriculture and the environment. Below, we review key changes in the revised regulations, illustrate their application using examples of promising leads in the literature, and show how they are likely to spur trait development that hold promise for improving agricultural sustainability and addressing some climate challenges.

## Changes in USDA’s revised biotech regulation

Under the revised regulations, and using Plant Protection Act authority, USDA considers whether an organism developed using genetic engineering poses an increased plant pest risk relative to a suitable comparator (USDA-APHIS, 2020). Several key changes contribute to more risk-proportionate regulation for plants created using genetic engineering. First, the revised regulations establish three exemptions for certain modifications a plant developed using genetic engineering may contain.^1^

These exemptions are based on types of modifications that commonly occur during conventional breeding (USDA-APHIS, 2020; Hoffman, 2021). Basing the exemptions on specifically described modifications, rather than a risk assessment, allows developers to objectively assess whether their plants meet the criteria exemption.

Second, the revised regulations establish an exemption for a plant-trait-mechanism of action (MOA) combination that USDA previously reviewed and determined not to pose a plant pest risk. This provision eliminates the burden of unnecessary re-reviews of plants whose risks were already considered.^2^

Third, the revised regulations establish a risk-based approach called regulatory status review (RSR) to determine whether a non-exempt plant is regulated. A key difference between RSR and the previous petition process is that RSR uses problem formulation and risk assessment to evaluate the characteristics and risk of the plant prior to a formal determination to continue to exercise oversight (Hoffman, 2021). Under the revised regulation, a developer can elect to undergo the RSR process prior to field testing. USDA expects that the RSR process will be an effective means to winnow the potentially riskier plants developed using genetic engineering from the less risky ones without imposing substantial regulatory burdens on the less risky ones (Hoffman, 2021). Among other things, USDA considers whether a genetic modification to a plant will increase the likelihood of harm to non-target species beneficial to agriculture or will increase the distribution or exacerbate the impact of plant pests that may be associated with that plant. USDA will undertake an initial review to efficiently distinguish plants developed using genetic engineering that do not pose plausible pathways to increased plant pest risk from those that do and, thus, require further evaluation. This initial review, which is based on a description of the plant, the trait, and the MOA, does not initially require field data. When USDA finds a plant does not pose plausible pathways to increased plant pest risk during the initial review phase, developers can attain regulatory certainty early in product development, which helps developers raise venture capital to see a product through to launch.^3^

## Biotechnology crop development under the legacy regulation

In 2008-2012, the mean cost for discovery, development, and authorization of a new crop created using genetic engineering was $136 M, where regulatory requirement costs averaged $35.1M, and the mean duration to bring a crop to market was 13.1 years (McDougall, 2011). The high-cost developments and long duration reduce return on investment. Consequently, the technology has principally been used on large acreage crops (corn, soybean, cotton, alfalfa, sugarbeet, potato, and canola) engineered with relatively few traits. Of the 136 petitions granted non-regulated status by the USDA, 109 were from those 7 major crops, and 80 had no traits other than either herbicide or insect resistance^4^. Under the legacy regulations, not all crops created with genetic engineering fell under the regulations. In 2010, USDA instituted a process known as “Am I Regulated” (AIR process), which provided a voluntary mechanism for developers to obtain USDA’s opinion about whether a plant was subject to regulation^5^. During the last 10 years (2011-2020), the number of organizations using the AIR process increased nearly 4 fold relative to the petition process, while the number of different crops considered increased greater than 5 fold (Hoffman, 2021), suggesting the legacy regulation created a significant barrier to market development of any crop that fell under the regulations.

We expect that the new more risk-appropriate regulation will lead to the commercialization of additional crops and traits. Current literature shows hundreds of proofs of concept for traits with potential to meet SDGs. The examples we provide: exclude plants currently subject to regulation as most have associated Confidential Business Information claims; only include crop plants tested either in the field or greenhouse; and include one representative example when the same plant-trait-MOA was discussed in more than one paper. For each example, we considered whether the plant would likely qualify for an exemption (**Table 1**) or likely be evaluated under the RSR process (**Table 2**) based on the information in the paper (**Supplementary Table 1** provides additional information on the MOA). Importantly, these considerations are meant to give an approximation of traits relevant to sustainability goals that are ripe for development; they are not and should not be construed as regulatory decisions since we may be missing key details.

**Table 1:** Crops that may contribute to sustainable agriculture that could potentially qualify for USDA regulatory exemption.

Trait	Distinct MOAs	Crops	SDG
Better suitability for urban agriculture	1	tomato	2, 11, 15
breeding innovation	8	alfalfa, cabbage, corn, cucumber, potato, sorghum, tomato, wheat	1, 2, 9
domestication	2	canola, ground cherry	1, 2, 3, 9, 11, 15
improved nutrition	10	Cassava, pennycress, rice (4), soybean, tomato(3)	2, 3, 15
increased disease tolerance	14	apple, banana, barley, canola, cassava, corn, cucumber, rice (3), tomato, watermelon, wheat (2)	1, 2, 13, 15
increased tolerance of abiotic stress	4	rice (4)	1, 2, 13, 15
increased yield	11	corn, rice (7), soybean, tomato, wheat	1, 2, 11, 15
reduced postharvest losses	3	tomato (3)	1, 2, 11, 12, 15
			
increased yield and increased tolerance to abiotic stress	2	rice (2)	2, 13, 15
improved nutrition increased disease tolerance	1	soybean	2, 3, 15
increased yield and reduce fertilizer requirement	1	rice	2, 13, 14, 15
increased yield, better suitability for urban agriculture	5	Tomato (4), ground cherry	2, 3, 11, 13, 15
increased yield and increased tolerance to abiotic stress and reduced fertilizer requirement	1	rice	2, 13, 15
Total	63		

**Table 1** represents a tally of all the plant-trait MOAs listed in **Supplementary Table 1** that could potentially qualify for exemption under USDA’s revised biotechnology regulation. Plant-Trait-MOAs for representative cases from the literature where crops could contribute to UN sustainable goal are listed in **Supplementary Table 1**. In each case, a high-level category corresponding to column 1 of this table was assigned, as was the SDGs that could be impacted by the launch and adoption of that crop. Column 2 lists the distinct number of MOAs responsible for the phenotype categorized in column 1. The UN sustainability goals listed in the table are as follows:

Sustainability Development Goals (SDG)

#1 ending poverty

#2 ending hunger

#3 good health

#9 industry innovation and infrastructure

#11 resilient and sustainable cities

#12 sustainable consumption and production

#13 climate action

#14 life below water

#15 life on land

Currently the exemptions under the revised regulation allow a single modification to a pair of homologous chromosomes per life cycle. Multiple modifications based on stacking traits through conventional breeding qualify for the exemption, but molecular stacks do not. Therefore, a single change to all homoeologous alleles in a polyploid presently do not qualify for the exemption.^6^It should be noted that there are opportunities to expand the exemptions when new information emerges demonstrating such modifications are possible by conventional breeding.^7^

**Table 2:** Crops that could contribute to sustainable agriculture that would undergo regulatory status review.

Trait	Distinct MOAs for RSR	Crops	SDG
breeding innovation	2	canola, rice	1, 2, 9, 15
domestication	3	canola, kiwi, wild tomato	1, 2, 3, 15
improved nutrition	18	banana, camelina, canola (4), cotton (3), potato (2), sorghum sugarcane tomato (3), wheat (2)	2, 3, 14, 15
increased pest tolerance	11	canola, citrus, cotton, rice (2), soybean, tomato (2), wheat (3)	1, 2, 13, 15
increased tolerance of abiotic stress	19	banana, barley, canola, corn (2), poplar, rice (5) soybean, tobacco (2), tomato, wheat (4)	1, 2, 13, 15
increased yield	6	canola (3), soybean, tef, tobacco	1, 2, 13, 15
reduced fertilizer requirement	2	rice, tomato	1, 2, 6, 13, 14, 15
improved nutrition, reduced postharvest loss	1	tomato	1,2,3,11,12,15
improved nutrition and net zero aviation fuel	1	pennycress	3, 6, 7, 13, 14, 15
increased yield and increased quality	1	rice	1, 2, 3, 15
Increased yield and increased disease tolerance	1	rice	1, 2, 15
increased yield and increased tolerance to abiotic stress	14	corn (2), cotton (2), rice (7), potato, wheat (2)	1, 2, 13, 15
increased yield and reduce fertilizer requirement	2	rice, wheat	1, 2, 6, 13, 14, 15
Total	80		

**Table 2** represents a tally of all the plant-trait MOAs listed in **Supplementary Table 1** that would likely be evaluated by the RSR process under USDA’s revised biotechnology regulation at 7 CFR part 340. Plant-Trait-MOAs for representative cases from the literature where crops could contribute to UN sustainability goals are listed in **Supplementary Table 1**. In each case, a high-level category corresponding to column 1 of this table was assigned as was the SDGs that could be impacted by the launch and adoption of that crop. Column 2 lists the distinct number of MOAs responsible for the phenotype categorized in column 1. The UN sustainability goals listed in the table are as follows:

Sustainability Development Goals (SDG)

#1 ending poverty

#2 ending hunger

#3 good health

#6 clean water

#7 affordable clean energy

#9 industry innovation and infrastructure

#13 climate action

#14 life below water

#15 life on land

## Traits that may promote sustainable agriculture and/or mitigate adverse impacts of climate change

### Producing more with fewer resources

Producing more food with fewer resources directly supports SDGs associated with reducing poverty, ending hunger, climate action, and conservation (SDGs #1, #2, #6, #13, and #15).

Knockout (KO) strategies in corn, rice, soybean, tomato, and wheat have been used to delete quantitative trait loci (QTL) negatively associated with yield (Zhou et al., 2019), a subfamily of ABA receptors (Miao et al., 2018), or genes that change plant architecture to allow denser planting (Tian et al., 2019), changes in spike inflorescence architecture (Wang et al., 2022), simultaneous increases in panicle number and tiller number (Song et al., 2022; Huang et al., 2018), timing of flowering (Cai et al., 2020), or result in more fruit and less shoot per plant (Rodríguez-Leal et al., 2017). Such plants are likely exempt from regulation and have been shown to increase yield in initial studies without increasing inputs.

USDA would evaluate crops developed with transgenic modifications under the RSR process, as in the case of tobacco engineered for increased production through reduced photorespiration (Cavanagh et al., 2022) or acceleration of the relaxation of non-photochemical quenching during sun-shade transitions (De Souza et al., 2022). Likewise, KOs of all homoeologous alleles in polyploids that increase production would be evaluated under the RSR process, as in the case of canola (Yang et al., 2018; Karunarathna et al., 2020; Zheng et al., 2020) and tef (Beyene et al., 2022). Diploid rice with a KO of 3 different cytochrome P-450 genes to increase production (Usman et al., 2020b) may qualify for exemption depending on whether the individually exempt traits are stacked by breeding (which could be exempt) or molecularly (which would require RSR). Overexpression of a transcription factor in rice was shown to increase both yield and resistance to blight by Xanthomonas (Liu et al., 2019).

### Reduced postharvest losses

The Food and Agriculture Organization (FAO) estimates that 14% of the world’s food is lost from production before reaching the retail level (FAO, 2019). Reducing postharvest losses could increase food availability and could free land for conservation (SDGs #12, #2, #15). Developers have used genome editing to increase a tomato’s shelf life by either KO of pectate lyase (Uluisik et al., 2016), polygalacturonse (Nie et al., 2022) or by recreating the allele of a known delayed fruit deterioration mutation (Yu et al., 2017). Each of these tomato varieties would likely qualify for exemption.

### Increased disease tolerance

FAO estimates that annually 20-40 percent of global crop production is lost to pests (FAO, 2019). Increasing disease tolerance could result in increased food and could reduce the volume of crop protection chemicals applied for disease control supporting SDGs related to protecting human health, the land, and water related ecosystems (SDGs #2, #3, #15, #6).

Pathogens often exploit susceptible plant genes to facilitate their infection (Van Schie and Takken, 2014). KOs have been used on a variety of susceptible genes to increase disease tolerance in apple (Pompili et al., 2020), barley (Hoffie et al., 2022), canola with the susceptibility gene only in the A genome (Pröbsting et al., 2020), cassava (Gomez et al., 2019), corn (Liu et al., 2022), cucumber (Chandrasekaran et al., 2016), rice (Zhou et al., 2018), tomato (Nekrasov et al., 2017), and watermelon (Zhang et al., 2020a) (**Table 1**). Increased resistance to wheat stem rust Ug99 was conferred into wheat (*T. aestivum*) by introducing resistance genes from either einkorn wheat (*T. monococcum*) (Chen et al., 2018) or durham wheat *T. turgidum* (Zhang et al., 2017b), both of which are in the wheat gene pool. These examples and others in banana, rice and tomato (Tripathi et al. 2019; Macovei et al., 2018; Ortigosa et al., 2019; Zhou et al 2022b) would likely be exempt

In other instances, developers have used strategies that involve multiple modifications or the use of transgenes to increase disease resistance, which USDA would likely evaluate under the RSR process. KOs of susceptibility genes in canola (Sun et al., 2018), citrus (Peng et al., 2017), cotton (Zhang et al., 2018b) and wheat (Zhang et al., 2017c; Koller et al., 2019; Li et al., 2022b) conferred resistance to various diseases. Multiple promoter deletions in several sugar transport genes conferred broad spectrum resistance in rice to several races of bacterial blight (Oliva et al., 2019). With respect to transgenes, broad resistance to three rice diseases was accomplished by transcriptional and translational controlled expression of the *Arabidopsis* NPR1 gene (Xu et al., 2017). Expression of transgenes to confer host induced gene silencing has shown promise in controlling fungi, nematodes (Kong et al., 2022), sap sucking and chewing insects, and viruses (Koch and Wassenegger, 2021) and Crispr-Cas lines targeting virus coat proteins have conferred resistance to viruses (Tashkandi et al. 2018).

### Increased tolerance of abiotic stress

Climate change is expected to have a net negative impact on agricultural productivity (Raza et al., 2019). Traits that could help crops adapt to climate change include tolerance to heat, drought, and salinity.

**Table 1** lists 4 cases for increased abiotic tolerance, all in rice, that would likely qualify for an exemption based on partial deletions (Liu et al., 2020a; Santosh Kumar et al., 2020) or KO (Zhang et al., 2019) of endogenous transcription factors or addition of an allele of Sub1, a transcription factor within the rice gene pool that confers tolerance to flooding (Xu et al., 2006). KO of an ABA receptor increased both yield and tolerance to drought (Usman et al., 2020a) and a triple KO of a putative auxin transport protein, a QTL negatively associated with yield, and a MYB30 transcription factor increased both yield and cold tolerance (Zeng et al., 2019).

Tolerance to environmental stress has been conferred by overexpression of transcription factors that turn on genes in the stress response (Casaretto et al., 2016). Other strategies with successful proof of concept include reducing stomatal opening (Głowacka et al., 2018), increasing specific protein turnover (Alfatih et al., 2020), overexpressing glutaredoxins (Sprague et al., 2022), and modulating stress related signaling (Zang et al., 2018). In all these cases, crops yield better than the comparator under abiotic stress. In some cases, the modified plants yield better than controls even in the absence of stress (Beznec et al., 2021; Esmaeili et al., 2021; Yu et al., 2021; Sprague et al., 2022). Examples likely requiring an RSR are included in **Table 2** representing 27 cases from banana (Sreedharan et al., 2013), barley (Hughes et al., 2017), canola (Wu et al., 2020), corn (Shi et al., 2017; Nuccio et al., 2015), cotton (Mishra et al. 2017), poplar (Li et al., 2018b), potato (Yu et al., 2021), rice (Shim et al., 2018; El-Esawi and Alayafi 2019; Liu et al. 2020b; Caine et al., 2019; Zhang et al., 2018a; Jiang et al., 2019; Park et al., 2020; Joshi et al., 2019; Selvaraj et al. 2017; Selvaraj et al., 2020, Usman et al., 2020a), soybean (Ribichich et al., 2020), tobacco (Sharma et al., 2019), tomato (Yin et al. 2018), and wheat (El-Esawi et al., 2019; Zhang et al., 2017a; Mega et al., 2019; Zhou et al. 2022a; González et al., 2019).

### Reduced fertilizer requirement

Fertilizer costs are among the most expensive inputs for a farmer (Langemeier et al., 2019) and they represent one of the largest energy expenditures for agriculture (Amenumey and Capel, 2014). Agriculture nutrient runoff is a significant contributor to impairment in assessed rivers and streams (US-EPA, 2018), and the single largest source of nutrient pollution to the Gulf of Mexico’s “dead zone” (Ribaudo et al., 2011). Unabsorbed nitrogen by crops leads to increased production of nitrous oxide, a greenhouse gas which is 300 times more potent than carbon dioxide (Sisharmini et al., 2019). Crops that require less fertilizer input could contribute to clean water and streams, climate action, life below water, life on land, and to ending poverty by reducing fertilizer costs (SDGs #6, #13, #15, #1). Expression of rice GR4 transcription factor from its own promoter results in increases in nitrogen use efficiency (NUE) and yield (Li et al., 2018c). Rice with C-terminal indels in the gene lonely guy (OsLOG5), which catalyzes the formation of active cytokinin from inactive forms, led to increased yields under well-watered, drought, normal nitrogen and low nitrogen (Wang et al., 2020). Both may qualify for exemption.

Examples likely to require RSR evaluation include increased NUE in rice by expression of a cucumber alanine aminotransferase under the control of a rice root specific promoter (Sisharmini et al., 2019), increased NUE in rice through co-overexpression of the rice nitrate transporter (OsNRT2.3a) and its partner protein (NAR2.1a) under the control of the CaMV35S promoter (Chen et al., 2020), increased tolerance to potassium deficiency in rice by overexpression of a rice peroxiredoxin gene (Mao et al., 2018), and increased tolerance to phosphate deficiency in tomato by expression of choline oxidase from the bacteria, *Arthrobacter globiformis* (Li et al., 2019).

### Improved nutrition

Increasing the nutritional value of crops is consistent with good health and well-being (SDG #3) and has been accomplished through KO strategies likely qualifying for exemption in diploids and RSR in polyploids. Healthier fatty acids have been made in oil crops (eg. (Haun et al., 2014; Jarvis et.al, 2021; Okuzaki et al. 2018; Jiang et al., 2017). Other improvements in nutrition include low reducing sugars (potato) (Clasen et al., 2016), high amylose (rice) (Sun et al., 2017), high gamma aminobutyric acid (tomato) (Nonaka et al., 2017), increased vitamin D3 (tomato) (Li et al., 2022a), increased ascorbic acid (tomato) (Do et al., 2022; Zheng et al., 2022), high isoflavone (soybean) (Zhang et al., 2020b), high carotenoid (banana, rice, and tomato) (Paul et al., 2017; Li et al., 2018d), increased protein (canola) (Xie et al., 2020; Zhai et al., 2020), high anthocyanin (tomato) (Butelli et al., 2008), and high iron (Wheat) (Connorton et al., 2017).

In some cases, the crop was made healthier by reducing an anti-nutrient. KO of key transporter proteins resulted in plants that had low uptake of cadmium (Tang et al., 2017) or cesium (Nieves-Cordones et al., 2017). Cotton seed was engineered to be gossypol free thereby creating a new food source by making the meal and oil suitable for human and animal consumption (Sunilkumar et al., 2006; Janga et al., 2019; Li et al., 2021). Other toxic substances eliminated or reduced from crops include steroidal glycosides in potato (Nakayasu et al., 2018), erucic acid in pennycress (Mcginn et al., 2019), lignin in sugarcane (Kannan et al., 2018), kafirin (a difficult protein to digest) in sorghum (Li et al., 2018a), reduced phytic acid in canola (which increases the bioavailability of phosphate in feed) (Sashidhar et al., 2020), reduced cyanide in cassava (Juma et al., 2022), and reduced amylose (rice) and reduced gluten wheat which is of benefit to some on restricted diets (Sánchez-León et al., 2018; Yunyan et al., 2019).

### Domestication

Domestication of wild crops through centuries of breeding and selection has inadvertently reduced genetic diversity (Smýkal et al., 2018), limiting traits beneficial for sustainable agriculture. With an understanding of domestication traits, genome editing can rapidly improve agronomic performance of wild relatives by reuniting lost but desirable traits, such as stress tolerance, with agronomically valuable characteristics (Zsögön et al., 2018). For example, in a wild relative of tomato, an initial attempt has been made to make a new tomato variety by introducing six domestication traits that resulted in improvements in fruit number, size, shape, nutrient content and plant architecture (Zsögön et al., 2018). Similar examples are seen in alternative crops such as pennycress (Mcginn et al., 2019), ground cherry (Lemmon et al., 2018) and kiwi (Varkonyi-Gasic et al., 2019), and in standard crops such as canola and tomato (Braatz et al., 2017; Zhai et al., 2019; Kwon et al., 2020). The KO strategies used in (Lemmon et al., 2018; Zhai et al., 2019; Kwon et al., 2020) might qualify for exemption while the RSR process is more likely for the others named above.

### Breeding innovations

Hybrid corn seed is almost exclusively grown in the U.S. because of its dramatic increases in yield and vigor (Nielsen, 2020). Widespread use of hybrid seed technology occurred first in corn because the crop’s separate male and female flowers simplifies the hybridization procedure. In crops where hybrid seed is not economically viable because flowers have both male and female organs (perfect flowers), yield gains have typically languished relative to hybrid seed crops (Perez-Prat and Van Lookeren Campagne, 2002). In crops with perfect flowers, KO strategies have been used to introduce male sterility and/or eliminate self-incompatibility (Li et al., 2016; Li et al., 2017; Ye et al., 2018; Ma et al., 2019; Okada et al., 2019; Dai et al., 2022; Ye et al., 2022; Chen et al., 2019; Cigan et al., 2017) to enable efficient hybrid seed production. All female plants were produced in cucumber by KO of a gene required for carpel development (Hu et al., 2017) all of these would likely qualify for exemption. Potato, normally a tetraploid, is being reinvented into a diploid inbred line-based crop that will help achieve yield and vigor gains seen in other hybrid crops (Jansky et al., 2016; Hosaka and Sanetomo, 2020). Recently, apomixis was engineered in rice (Khanday et al., 2019). This trait will enable hybrid seeds to be propagated clonally thereby dramatically reducing the cost of hybrid seed. It involves the KO of three genes and the expression of a normally pollen-specific gene in the egg cell so it would be evaluated under the RSR process. These outcomes contribute to industry innovations (SDG #9) and are expected to facilitate the development of new crops with increased yields and tolerance to abiotic and biotic stresses consistent with numerous other SDGs.

### Urban agriculture

Urban vertical farming has been touted as a means to increase agricultural sustainability through demonstrated increases in agricultural productivity, food safety, biosecurity and reduced inputs (water, fertilizer, and pesticides), land use, and transportation costs (SDG #11) relative to outdoor agriculture (Benke and Tomkins, 2017) (SDGS #2, #3, #6, #11, #13-15). Using controlled environments with recycling of nutrients and water, renewable energy, and automation, vertical farming may also offer solutions to climate change and labor shortages that have plagued outdoor agriculture.

Genome editing has been used to create tomato varieties more suitable to controlled environment production (Klap et al., 2017; Soyk et al., 2017; Ueta et al., 2017; Tomlinson et al., 2019; Kwon et al., 2020). The tomato varieties described in the urban agriculture section would likely be eligible for exemption provided the traits were combined by breeding.

### Discussion

In this paper, we provide over 140 examples of crops that were created with biotechnology that could contribute to UN sustainability goals. This survey represents just a fraction of the traits being developed to improve sustainability and/or other purposes. Furthermore, traits that show promise in one species frequently prove to be valuable in improving closely related species. Considering that there are hundreds of crops related to those in **Tables 1** and **2** (Khoshbakht and Hammer, 2008), it implies that tens of thousands of new crop varieties can be created based on the examples listed in **Tables 1** and 2 alone. We estimate that over 60 of the described crop varieties would likely qualify for exemption from USDA oversight. For the remaining crops that would likely be evaluated through the RSR process, the regulatory pathway will be more risk-proportionate, science-based, product-based and streamlined compared to the former petition process (Hoffman, 2021). We already see academics interested in developing products that could successfully navigate the revised regulations. For example, although the Martin lab published the successful creation of a high anthocyanin tomato in 2008 (Butelli et al., 2008), they did not seek regulatory approval in the United States until April 2021 when the RSR process became first available for tomatoes and they received regulatory clearance September 2022.

Based on the large number of promising crop-trait-MOA combinations that have been discovered and the more streamlined, risk-proportionate, and science-based oversight in USDA’s revised regulation, we fully expect to see diverse developers advance more traits that may help promote sustainability. Whether they are ultimately commercialized may depend on overcoming other obstacles including requirements from other regulatory authorities, social understanding and acceptance, and on their economic viability. Both United States Food and Drug Administration (FDA) and Environmental Protection Agency (EPA) continue to play a role in the approval of biotechnology crops in the United States and are actively considering how to streamline their regulations regarding genome editing (OSTP, 2017). EPA has proposed an exemption for certain plant incorporated protectants created through biotechnology that could have otherwise been created through conventional breeding (US-EPA, 2012), while FDA has produced a plant and animal biotechnology innovation action plan and intends to update existing procedures for voluntary premarket consultations (US-Food and Drug Administration, 2018). Several countries (Brazil, Columbia, Argentina, Chile, Israel, Australia, and Japan) do not regulate some genome edited crops lacking foreign DNA as Genetically Modified Organisms (GMOs) and several other countries are considering adopting a similar approach (Schmidt et al., 2020). As the number of like-minded countries grow, regulatory obstacles are expected to diminish.

## Author contributions

The author confirms being the sole contributor of this work and has approved it for publication.

## Funding

This work was supported by the U.S. Department of Agriculture, Animal and Plant Health Inspection Service.

## Acknowledgments

The author gratefully acknowledges the critical review of the manuscript and valuable feedback by Bernadette Juarez, Alan Pearson, and Subray Hegde.

## Conflict of interest

The author declares that the research was conducted in the absence of any commercial or financial relationships that could be construed as a potential conflict of interest.

## Publisher’s note

All claims expressed in this article are solely those of the authors and do not necessarily represent those of their affiliated organizations, or those of the publisher, the editors and the reviewers. Any product that may be evaluated in this article, or claim that may be made by its manufacturer, is not guaranteed or endorsed by the publisher.

## Footnotes

1 The SECURE rule (15) exempts plants containing a single modification where 1) “the genetic modification is a change resulting from cellular repair of a targeted DNA break in the absence of an externally provided repair template; or 2) the genetic modification is a targeted single base pair substitution; or 3) the genetic modification introduces a gene known to occur in the plant’s gene pool or makes changes in a targeted sequence to correspond to a known allele of such a gene or to a known structural variation present in the gene pool.

2 Developers can request a confirmation from APHIS that a modified plant qualifies for an exemption and is not subject to the regulations in 7 CFR part 340. USDA APHIS | Confirmation Letters accessed 09.27.22

3
USDA APHIS | Plant-Trait-Mechanism of Action (MOA) combinations that have been determined by APHIS not to require regulation under 7 CFR part 340.accessed 09.27.22. USDA APHIS | Confirmation Letters accessed 09.27.22

4

USDA APHIS | Petitions for Determination of Nonregulated Status accessed 09.27.22

5

USDA APHIS | Regulated Article Letters of Inquiry accessed 09.27.22

6

q-a-confirmation-process.pdf (usda.gov) p.3-4.

7 CFR part 340.1(b)(4)
