# Supplementary material for: USDA’s revised biotechnology regulation’s contribution to increasing agricultural sustainability and responding to climate change
Source: Front Plant Sci. 2022 Nov 23;13:1055529. doi: 10.3389/fpls.2022.1055529 (PMC9726801; doi:10.3389/fpls.2022.1055529)
Supplement: Supplementary file 1 [file Table_1.docx]

## Supplementary Material

## TABLE S1|. Plant-Trait-Mechanism of Action (MOA) for examples used in TABLE 1 and TABLE 2.

| SDG | consolidated trait | Potential Regulatory Pathway^[[1]](#footnote-1)^ | Crop | Trait | MOA | Citation |
| --- | --- | --- | --- | --- | --- | --- |
| 2, 11, 15 | better suitability for urban agriculture | Exempt | tomato | dwarf phenotype | heterozygous or homozygous 3 nt deletion in PROCERA gene encoding DELLA protein | (Tomlinson et al., 2019) |
| 1,2,9 | breeding innovation | Exempt | alfalfa | male sterility | KO of No Pollen 1 (MsNP1), a putative glucose-methanol-choline (GMC) oxidoreductase | (Ye et al., 2022) |
| 1, 2, 9 | breeding innovation | Exempt | cabbage | suppression of self-incompatibility and male sterility for reducing the cost of hybrid seed production | KO of S-RECEPTOR KINASE (loss of self-incompatibility) and KO of MALE STERILE 1 (male sterility) | (Ma et al., 2019) |
| 1, 2, 9 | breeding innovation | Exempt | corn | thermosensitive male sterility | Homozygous KO of thermosensitive genic male-sterile 5 (TMS5) | (Li et al., 2017) |
| 1, 2 | breeding innovation | Exempt | cucumber | gynoecious for improved hybrid production and higher yield | homozygous KO of WIP1 | (Hu et al., 2017) |
| 1, 2, 9 | breeding innovation | Exempt | potato | Self-compatibility to facilitate breeding in diploid potato | homozygous knockout of one S-RNAse gene | (Ye et al., 2018) |
| 1, 2 | breeding innovation | Exempt | rice | male sterility for capturing heterosis through hybrid seed | KO of *Carbon Starved Anther* | (Li et al., 2016) |
| 1, 2 | breeding innovation | Exempt | sorghum | male sterility for capturing heterosis through hybrid seed | homozygous KO of Ms26 (anther specific P450 gene) | (Cigan et al., 2017) |
| 1,2 | breeding innovation | Exempt | tomato | male sterility for capturing heterosis through hybrid seed | homozygous KO of Respiratory Burst Oxidase Homologue *(Slrbohe-1)* | (Dai et al., 2022) |
| 1, 2 | breeding innovation | Exempt | wheat | male sterility for capturing heterosis through hybrid seed | homozygous KO of one set of Ms1 alleles (homeologues are epigenetically silenced | (Okada et al., 2019) |
| 1, 2, 15 | domestication | Exempt | canola | reduced shattering | KO of INDIHISCENT (alleles on the A genome) | (Zhai et al., 2019) |
| 2, 3, 11 | domestication | Exempt | Ground-cherry | compact shoot growth, increased flower number, increased fruit size | KO of orthologues of self-pruning, self-pruning 5G, KO of clavata 1, | (Lemmon et al., 2018) |
| 2,3 | improved nutrition | Exempt | cassava | reduced cyanide | KO of cytochrome P450 (MeCYP79D1) which catalyzes the first step in cyanogenic glucoside biogenesis (linamarin and lotaustralin). | (Juma et al., 2022) |
| 3 | improved nutrition | Exempt | pennycress | high oleic acid | KO of REDUCED OLEATE DESATURATION1 (TaROD1) and FATTY ACID ELONGATION1 (TaFAE1) | (Jarvis et al., 2021) |
| 2, 3, 15 | improved nutrition | Exempt | rice | low cesium uptake and accumulation (desired in areas with radioactive soil waste) | homozygous KO of Cs+ permeable K+ transporter OsHAK1 | (Nieves-Cordones et al., 2017) |
| 3 | improved nutrition | Exempt | rice | reduced amylose | Homozygous KO of starch synthase Wx (waxy) | (Yunyan et al., 2019) |
| 3 | improved nutrition | Exempt | tomato | increase in gamma amino butyric acid | glutamate decarboxylase C-terminal truncation GAD3 gene | (Nonaka et al., 2017) |
| 3 | improved nutrition | Exempt | tomato | Increased vitamin D3, reduced tomatine | KO of 7-dehydrocholesterol reductase (SI7-DR2) | (Li et al., 2022a) |
| 3 | Improved nutrition | Exempt | tomato | Increased ascorbic acid | KO of Ascorbate Peroxidase 4 *(SLAPX4*) | (Do et al., 2022) |
| 3 | improved nutrition | Exempt | rice | high amylose | KO of STARCH BRANCHING ENZYME (SBEIIb) | (Sun et al., 2017) |
| 3 | improved nutrition | Exempt | rice | low cadmium accumulating | homozygous KO of natural resistance associated macrophage proteins (Nramp5) a metal transporter | (Tang et al., 2017) |
| 3 | improved nutrition | Exempt | soybean | high oleic acid | quadruple KO, FATTY ACID DESATURASE2 (FAD2-1A and FAD2-1B) | (Haun et al., 2014) |
| 2, 3, 15 | improved nutrition increased disease tolerance | Exempt | soybean | increased isoflavone content/increased resistance to soybean mosaic virus | Sextuple KO, flavanone-3-hydroxylase (F3H1, F3H2) and flavone synthase II (FNS II). | (Zhang et al., 2020b) |
| 1, 2, 15 | increased disease tolerance | Exempt | apple (varieties gala and golden delicious | reduced fire blight susceptibility | KO of DIPM4 | (Pompili et al., 2020) |
| 1, 2, 15 | increased disease tolerance | Exempt | banana | inactivation of banana streak virus integrated into banana germplasm | KO of BSV ORFs | (Tripathi et al., 2019) |
| 1,2,15 | increased disease tolerance | Exempt | barley | Increased resistance to barley yellow mosaic virus (BaYMV) and barley mild mosaic virus (BaMMV) | KO of PROTEIN DISULFIDE-ISOMERASE-LIKE 5-1 (PDIL5-1) | (Hoffie et al., 2022) |
| 1, 2, 15 | increased disease tolerance | Exempt | canola | increased resistance to Verticillium longisporum | KO of CALRETICULIN (CRT1) A genome | (Pröbsting et al., 2020) |
| 1, 2, 15 | increased disease tolerance | Exempt | cassava | reduced susceptibility to cassava brown streak (caused by cassava brown streak virus: CBSV and Uganda cassava brown streak virus: UCBSV | Homozygous KO of novel cap-binding protein-1 (nCBP-1 and nCBP-2) forms of translation initiation factor eIF4E. | (Gomez et al., 2019) |
| 1,2,15 | increased disease tolerance | Exempt | corn | Resistance to rice black-streaked virus (RBSDV) causing maize rough dwarf disease | KO of Rab-GDP-dissociation inhibitor α-isoform (ZmGDIα) which functions in vesicle-membrane transport to recycle and regulate Rab GTPases | (Liu et al., 2022) |
| 1, 2, 15 | increased disease tolerance | Exempt | cucumber | resistance to cucumber vein yellowing virus zucchini mosaic virus and papaya ring spot mosaic virus-W | homozygous KO of eIFAE | (Chandrasekaran et al., 2016) |
| 1, 2, 15 | increased disease tolerance | Exempt | rice | increased resistance to blight (*Magnaporthe oryzae*)) | KO of ERF922 (Ethylene Responsive Factor) | (Zhou et al., 2022b) |
| 1, 2, 15 | increased disease tolerance | Exempt | rice | increased resistance against *Magnaporthe oryzae* (blast) and *Xanthomonas oryzae* (blight) | homozygous KO of BSR-K1 (Tetratricopeptide Repeat protein, that regulates mRNA metabolism including the level of OsPAL mRNA-that confers immunity upon pathogen infection | (Zhou et al., 2018) |
| 1, 2, 15 | increased disease tolerance | Exempt | rice | increased resistance to Rice tungro disease (caused by Rice tungro spherical virus and Rice tungro bacilliform virus | in frame deletion of N1053L1054 in translation initiation factor 4 gamma gene (OseIF4G) | (Macovei et al., 2018) |
| 1, 2, 15 | increased disease tolerance | Exempt | tomato | increased resistance to bacterial speck disease | c-terminal truncation of SIJAZ2 gene, a jasmonate zim domain repressor protein involved in regulating stomatal aperture in response to the toxin coronatine. | (Ortigosa et al., 2019) |
| 1, 2, 15 | increased disease tolerance | Exempt | tomato | powdery mildew resistance | homozygous KO of MILDEW RESISTANT LOCUS (Mlo1) | (Nekrasov et al., 2017) |
| 1, 2, 15 | increased disease tolerance | Exempt | Water-melon | increased resistance to *Fusarium oxysporium* | KO of psk1 (involved in phytosulfokine signaling) | (Zhang et al., 2020a) |
| 1, 2, 13, 15 | increased disease tolerance | Exempt | wheat | increased resistance against stem rust Ug99 | expression of Sr21(stem rust resistance gene from einkorn wheat TmCNL1 an LRR protein (Leucine rich repeat) | (Chen et al., 2018) |
| 1, 2, 15 | increased disease tolerance | Exempt | wheat | increased resistance against stem rust Ug99 | expression of Sr13 (stem rust resistance gene from durum wheat, *Triticum turgidum*, Tt CNL13 an LRR protein | (Zhang et al., 2017b) |
| 1, 2, 15 | increased tolerance of abiotic stress | Exempt | rice | increased salinity tolerance | Homozygous KO of OsRR22 transcription factor | (Zhang et al., 2019) |
| 1, 2, 13,15 | increased tolerance of abiotic stress | Exempt | rice | flood tolerance | overexpression of rice Submergence 1 (Sub1-A) | (Xu et al., 2006) |
| 1, 2, 13,15 | increased tolerance of abiotic stress | Exempt | rice | tolerance to salinity and drought stress | Deletion of aa 184 to 305 in Drought and Salt Tolerance gene (OsDST) transcription factor | (Santosh Kumar et al., 2020) |
| 1, 2, 13,15 | increased tolerance of abiotic stress | Exempt | rice | increase heat tolerance | heat inducible expression of a NAC type transcription factor (NAM-NO APICAL MERISTEM), ATAF1 (Arabidopsis thaliana Activation Factor 1), and CUC2 (Cup shaped cotyledon), lacking transmembrane domain | (Liu et al., 2020a) |
| 1, 2 | increased yield | Exempt | corn | reduced leaf angle allowing higher density planting and higher yields | Homozygous KO of Related to ABI3/VP1RAV-Like1 (RAVL1) | (Tian et al., 2019) |
| 1, 2 | increased yield | Exempt | rice | increased yield (grain length, width, number, weight) | homozygous KO of three QTLs negatively associated with yield | (Zhou et al., 2019) |
| 1, 2 | increased yield | Exempt | rice | increased yield (grain #/panicle) | 3' frameshift deletion of Gn1a cytokinin oxidase/dehydrogenase | (Huang et al., 2018) |
| 1, 2, 15 | increased yield | Exempt | rice | increased yield | Homozygous KO of GRAIN SIZE 3 (OsGS3) QTL that may function in the ubiquitination pathway | (Usman et al., 2021) |
| 1, 2, 15 | increased yield | Exempt | rice | increased yield | Homozygous triple KO of PyraBACTIN RESISTANCE-LIKE 1, 4, and 6(OsPYL1; Os PYL4, Os Pyl6 (ABA receptor) | (Miao et al., 2018) |
| 1, 2 | increased yield | Exempt | rice | increased yield (panicle #/plant) | 3' frameshift deletion of phosphatidylethanolamine-binding protein | (Huang et al., 2018) |
| 1,2 | increased yield | Exempt | rice | Increased panicle number and increased tiller number | 54 bp deletion in the promoter of IPA1, encoding a SQUAMOSA promoter binding protein-like (SPL) transcription factor, OSSPL14, a master regulator of rice plant architecture. | (Song et al., 2022) |
| 1, 2 | increased yield | Exempt | soybean | delayed flowering under short days (allows increased yields in tropics) | KO, FLOWERLOCUST (FT) FT2a and FT5a | (Cai et al., 2020) |
| 1, 2, 11 | increased yield | Exempt | tomato | increase flora organ number or fruit size conferring enhanced fruit yield | quantitative variation by promoter mutations in signaling peptide, Clavata3, inflorescence architecture gene compound inflorescence (S), or sel- pruning (SP) for determinate growth | (Rodríguez-Leal et al., 2017) |
| 1,2, | increased yield | Exempt | wheat | Increased yield | KO of DUO1, an APETALA2/ethylene response factor, regulating spike inflorescence architecture. | (Wang et al., 2022) |
| 1, 2, 13,14, 15 | increased yield and reduced fertilizer requirement | Exempt | rice | increased nitrogen use efficiency, increased yield | expression of rice GR4 (transcription factor of multiple nitrogen-metabolism genes) from native promoter | (Li et al., 2018c) |
| 1, 2, 11, 13,15 | increased yield, better suitability for urban agriculture | Exempt | tomato | seedless fruit (parthenocarpic -fruit formation without pollination) | homozygous KO of IAA9 gene (negative auxin regulator involved in tomato fruit development) | (Ueta et al., 2017) |
| 1, 2, 3, 13, 15 | increased yield, better suitability for urban agriculture | Exempt | tomato | facultative parthenocarpy under heat stress | KO of AGAMOUS-LIKE6 | (Klap et al., 2017) |
| 1, 2, 11 | increased yield, better suitability for urban agriculture | Exempt | tomato | increased yield (increased branching, flowering, and fruit number) | heterozygous weak alleles of the MADS box protein, Sepallata 4 | (Soyk et al., 2017) |
| 11 | increased yield, better suitability for urban agriculture | Exempt | tomato, ground cherry | rapid cycling (early flowering), compact growth | homozygous triple KO of SELF PRUNING (SP) which leads to determinate growth; sp5g (which leads to early flowering); and ERECTA (ER) which causes short internodes. | (Kwon et al., 2020) |
| 1, 2, 13, 15 | increased yield, increased tolerance of abiotic stress | Exempt | rice | increased tolerance to drought, increased grain yield | Homozygous KO of PYRABACTIN RESISTANCE-LIKE 9(OsPYL9 ABA receptor) | (Usman et al., 2020a) |
| 1, 2, 13, 15 | increased yield, increased tolerance of abiotic stress | Exempt | rice | increased yield, tolerance to cold stress | Homozygous triple mutant KO of PIN-FORMED (OsPIN5b; putative auxin transport protein KO results in increased panicle length), Grain size 3 (GS3; QTL-KO results in large grain), OsMYB30 transcription factor, (KO confers cold tolerance) | (Zeng et al., 2019) |
| 1, 2, 13, 15 | increased yield, increased tolerance of abiotic stress, reduced fertilizer requirement | Exempt | rice | increase grain yield under well-watered, drought, normal nitrogen, and low nitrogen | C-terminal indels in lonely guy (OsLOG5) which catalyzes the formation of active cytokinin from inactive forms | (Wang et al., 2020) |
| 1, 2, 11 | reduced postharvest losses | Exempt | tomato | delayed softening for increased shelf life | homozygous KO of pectate lyase | (Uluisik et al., 2016) |
| 1, 2, 11,12, 15 | reduced postharvest losses | Exempt | tomato | reduction of postharvest losses | alcobaca allele recreated by single bp substitution of thymine 317 to adenine in elite tomato variety | (Yu et al., 2017) |
| 1,2,11 | reduced postharvest losses | Exempt | tomato | Delayed softening for increased shelf life | homozygous KO of polygalacturonase (*SlPG*) | (Nie et al., 2022) |
| 1, 2, 9, 15 | breeding innovation | RSR | canola | loss of self-incompatbility | quadruple KO of M-LOCUS PROTEIN Kinase | (Chen et al., 2019) |
| 1, 2, 9, 15 | breeding innovation | RSR | rice | clonal propagation of hybrids. | KO of REC8, PAIR1, OSD1 and expression of BBM1 in the egg cell | (Khanday et al., 2019) |
| 1, 2 | domestication | RSR | canola | reduced shattering | quadruple KO of Alcatraz (ALC) | (Braatz et al., 2017) |
| 2, 3 | domestication | RSR | kiwi | early flowering, compact growth, determinate flowering | KO of CEN4 and CEN | (Varkonyi-Gasic et al., 2019) |
| 2, 3, 15 | domestication | RSR | wild tomato *Solanum pimpinellifolium* | domestication through increased fruit size, oval shape, increased lycopene, reduced height, determinate growth, and increased fruit number | KO of self-pruning (reduced height, determinate growth), OVATE (oval fruit), CLAVATA3 (multilocular- fruit), lycopene beta cyclase (increased lycopene), Fruit weight 2.2 , MULT (increased fruit number), FAS and Fruit weight 2.2 (larger fruit) | (Zsögön et al., 2018) |
| 2, 3 | improved nutrition | RSR | banana | elevated carotenoid | overexpression of banana phytoene synthase2a under the control of maize ubiquitin promoter (Zm-Ubi1-MtPsy2a) | (Paul et al., 2017) |
| 3 | improved nutrition | RSR | camelina | high oleic acid | sextuple KO of FAD2 genes | (Jiang et al., 2017) |
| 3 | improved nutrition | RSR | canola | high oleic acid | quadruple KO of FAD2 | (Okuzaki et al., 2018) |
| 2, 15 | improved nutrition | RSR | canola | yellow seed coat, increased protein | quadruple KO of TRANSPARENT TESTA 8 (transcription factor) | (Zhai et al., 2020) |
| 2, 15 | improved nutrition | RSR | canola | yellow seed coat, increased protein | quadruple KO of TRANSPARENT TESTA 2 (transcription factor) | (Xie et al., 2020) |
| 2, 3, 14 | improved nutrition | RSR | canola | reduced phytic acid | sextuple KO of INOSITOL TETRAKISPHOSPHATE KINASE (itpk) | (Sashidhar et al., 2020) |
| 2, 3, 15 | improved nutrition | RSR | cotton | gossypol free | KO of 4 alleles of PIGMENT GLAND FORMATION (PGF) | (Li et al., 2021) |
| 2, 3, 15 | improved nutrition | RSR | cotton | reduced gossypol | RNAi to decrease expression of delta-cadinene synthase | (Sunilkumar et al., 2006) |
| 2, 3, 15 | improved nutrition | RSR | cotton | reduced gossypol | KO of 4 alleles of PIGMENT GLAND FORMATION (PGF) and or COTTON GLAND FORMATION2 CGF2 | (Janga et al., 2019) |
| 3, 6, 7, 13,14, 15 | improved nutrition and net zero aviation fuel | RSR | pennycress | low erucic acid and altered oil for diesel engine drop in fuel | KO of *Thlaspi arvense* FATTY ACID ELONGATION(FAE1) and expression of *Euonymus alatus* diacylglycerol acetyltransferase (EaDAcT) | (McGinn et al., 2019) |
| 3 | improved nutrition | RSR | potato | reduced acrylamide, reduced sugars | Quadruple knockout of vacuolar invertase genes (*Vlnv)* | (Clasen et al., 2016) |
| 3 | improved nutrition | RSR | potato | steroidal glycoside free (alpha solanine and alpha chaconine free) | quadruple KO of 2-oxoglutarate-dependent dioxygenase (16DOX) | (Nakayasu et al., 2018) |
| 2, 3 | improved nutrition | RSR | sorghum | increased protein quality and digestibility | KO of eleven alleles of alpha Kafierin (k1C) | (Li et al., 2018a) |
| 13 | improved nutrition | RSR | sugarcane | reduced lignin | KO of 107 alleles of caffeic acid o-methyltransferase (COMT) | (Kannan et al., 2018) |
| 3 | improved nutrition | RSR | tomato | high anthocyanin | Snapdragon transcription factors Delila (Am Del) and Rosea1 (Am Ros1) under the control of fruit specific E-8 promoter from tomato (SlE8p) | (Butelli et al., 2008) |
| 3 | improved nutrition | RSR | tomato | Increased ascorbic acid | Overexpression of tomato dehydroascorbate reductase (*SiDHAR*) under control of CaMV35S promoter | (Zheng et al., 2022) |
| 2, 3, | improved nutrition | RSR | tomato | increased lycopene | homozygous KO of 4 genes, Stay Green 1, lycopene cyclase E, lycopene cyclase B2, beta lycopene cyclase | (Li et al., 2018d) |
| 3 | improved nutrition | RSR | wheat | low gluten | KO of 35 alpha gliadin genes | (Sánchez-León et al., 2018) |
| 3 | improved nutrition | RSR | wheat | increased iron | overexpression of VACUOLAR IRONH TRANSPORTER 2 (TaVIT2) | (Connorton et al., 2017) |
| 1,2,3,1112,15 | improved nutrition, reduced postharvest loss | RSR | tomato | Increased ascorbic acid, delayed fruit softening, enhanced tolerance to *Bortrytis cinerea* | Overexpression of tomato ionositol monophosphatase (SiIMP3) under the control of the CaMV35S promoter | (Zheng et al., 2022) |
| 1, 2, 15 | increased disease tolerance | RSR | canola | enhanced sclerotinia resistance | KO of three of 6 copies of WRKY70 transcription factor | (Sun et al., 2018) |
| 1, 2, 15 | increased disease tolerance | RSR | citrus | increased resistance to citrus canker disease | quadruple KO of lateral organ boundaries 1l | (Peng et al., 2017) |
| 1, 2, 15 | increased disease tolerance | RSR | cotton | increased resistance to cotton verticillium wilt (caused by *Verticillium dahliae*) | KO of all 4 alleles of 14-3-3d (negative regulators of brassinosteroid signaling) | (Zhang et al., 2018b) |
| 1, 2, 15 | increased disease tolerance | RSR | rice | broad spectrum resistance to bacterial blight | multiple promoter deletions in three sucrose transporter genes, SWEET11, SWEET13, and SWEET14 | (Oliva et al., 2019) |
| 1, 2, 13 15 | increased disease tolerance | RSR | rice | broad spectrum disease resistance to Rice blight (from *Xanthomonas oryzae pv. Oryzae*), rice bacterial leaf streak (from *X oryzae pv*. *oryzicola)*, and fungal blast (from *Magnaporthe oryzae*) | Transcriptional and translational controlled expression of AtNPR1 (NONEXPRESSER of PR (pathogenesis related) GENES1 using the uORF (upstream open reading frames) and promoter of TL1-binding factor (TBF1, a transcription factor that induces the immune response in plants)(AtTBF1p::uORFs_TBF1_AtNPR1) | (Xu et al., 2017) |
| 1,2,15 | increased disease tolerance | RSR | soybean | Increased resistance to soybean cyst nematode caused by *Heterodera glycines* and Fusarium wilt disease caused by *F. oxysporum*. | Host induced gene silencing using a chitin synthase from *H. glycines* under the control of the CaMV35S promoter. | (Kong et al., 2022) |
| 1, 2, 15 | increased disease tolerance | RSR | tomato | resistance to tomato yellow leaf curl virus | stable CRISPR/Cas9 line targeting TYLCV coat protein | (Tashkandi et al., 2017) |
| 1, 2, 15 | increased disease tolerance | RSR | wheat | increased resistance to powdery mildew | sextuple KO of Mildew Resistance Locus genes (MLO) including a 304 kb deletion on chromosome 4B which leads to activation of *Tonoplast monosaccharide transporter 3* (*TaTMT3B*) | (Li et al., 2022b) |
| 1, 2, 15 | increased disease tolerance | RSR | wheat | resistance to powdery mildew | sextuple KO of ENHANCED DISEASE RESISTANCE 1 (EDR1) | (Zhang et al., 2017c) |
| 1, 2, 15 | increased disease tolerance | RSR | wheat | powdery mildew resistance | Corn ubi1 driven expression of wheat Pm3e (ZmUBI1::TaPm3e; nucleotide binding leucine rich repeat (NLR)type of immune receptor | (Koller et al., 2019) |
| 1, 2, 15 | increased yield, increased disease tolerance | RSR | rice | increased yield, increased resistance to blight by *Xanthomonas* | increased expression of Ideal Plant Architecture1 (IPA1) transcription factor under the control of the pathogen inducible OsHEN1 promoter OsHEN1::OsIPA1 | (Liu et al., 2019) |
| 1,2,13, 15 | increased tolerance of abiotic stress | RSR | banana | Increased tolerance to drought, cold, and salinity (faster recovery) | Constitutive/conditional overexpression of banana plasma membrane intrinsic protein (*MusaPIP1;2*), an aquaporin involved in water homeostasis, under the control of the corn polyubiquitin promoter-*ZmUbi* or the stress inducible banana dehydrin promoter (Musa*Dhn*) | (Sreedharan et al., 2013) |
| 1, 2, 13,15 | increased tolerance of abiotic stress | RSR | barley | increased tolerance to drought, increased water use efficiency through reduced stomatal density | Overexpression of barley EPIDERMAL PATTERNING FACTOR 1 (HvEPF1) using CaMV35S promoter | (Hughes et al., 2017) |
| 1, 2, 13,15 | increased tolerance of abiotic stress | RSR | canola | tolerant to drought stress | Gain of Function in frame deletion in della and TVHYNP domains of REPRESSOR OF ga1-3 (RGA) BnA6.RGA transcription factor | (Wu et al., 2020) |
| 1, 2, 13, 15 | increased tolerance of abiotic stress | RSR | corn | increased yield under water stress | replacement of ARGOS8 promoter with a maize GOS2 promoter to increase expression | (Shi et al., 2017) |
| 1, 2, 15 | increased tolerance of abiotic stress | RSR | corn | improved drought and heat tolerance | Overexpression of rice MYB55 | (Casaretto et al., 2016) |
| 13, 15 | increased tolerance of abiotic stress | RSR | *Populus trichocarpa* | increased drought tolerance | overexpression of three transcription Factors PtNAC006, PtNAC007, and PtNAC120 | (Li et al., 2018b) |
| 1, 2, 13, 15 | increased tolerance of abiotic stress | RSR | rice | increased heat tolerance | overexpression of rice OsNAC14 transcription factor under control of the rice phosophogluconate dehydrogenase 1 (OsPGD1::OsNAC14) | (Shim et al., 2018) |
| 1, 2, 13,15 | increased tolerance of abiotic stress | RSR | rice | increased tolerance to oxidative and salt stress | homozygous KO of PARAQUAT TOLERANCE 3 (E3 ubiquitin lyase) | (Alfatih et al., 2020) |
| 1, 2, 13,15 | increased tolerance of abiotic stress | RSR | rice | tolerance to salinity | Overexpression of GT-gamma-2 transcription factor OsGTgamma-2 | (Liu et al., 2020b) |
| 1, 2, 13,15 | increased tolerance of abiotic stress | RSR | rice | increased drought tolerance | Overexpression of rice EPIDERMAL PATTERNING fACTOR1 (OsEPF1 signaling peptide) which reduces stomatal density | (Caine et al., 2019) |
| 1, 2, 13, 15 | increased tolerance of abiotic stress | RSR | rice | increased drought tolerance | knockdown of rice microRNA166 by short tandem target mimic or overexpression of HOMEODOMAIN CONTAINING PROTEIN 4) (OsHB4) | (Zhang et al., 2018a) |
| 1, 2, 13,15 | increased tolerance of abiotic stress | RSR | rice | increased drought and salinity tolerance | Overexpressing a microRNA (miR164b) resistant Os NAC2 | (Jiang et al., 2019) |
| 1, 2, 13,15 | increased tolerance of abiotic stress | RSR | soybean | increased drought and heat tolerance | sunflower homeodomain-leucine zipper transcription factor (HB4) under the control of its own promoter HaHB4) | (Ribichich et al., 2020) |
| 1, 15 | increased tolerance of abiotic stress | RSR | tobacco | increased salt and drought tolerance | Overexpression of apple DEHYDRATION RESPONSIVE ELEMENT BINDING PROTEIN (MdDREB76) transcription factor | (Sharma et al., 2019) |
| 1, 2, 13, 15 | increased tolerance of abiotic stress | RSR | tobacco | increased water use efficiency | Overexpression of tobacco PsbS driven by CaMV35S promoter | (Głowacka et al., 2018) |
| 1, 2, 13,15 | increased tolerance of abiotic stress | RSR | tomato | increased heat tolerance | overexpression of tomato BRASSINAZOLE RESISTANT 1 (BZR1) transcription factor that regulates BR responsive genes | (Yin et al., 2018) |
| 1, 2, 15 | increased tolerance of abiotic stress | RSR | wheat | improved drought and heat tolerance | Overexpression of Arabidopsis WRKY30 transcription Factor | (El-Esawi et al., 2019) |
| 1, 2, 13,15 | increased tolerance of abiotic stress | RSR | wheat | increased drought and heat tolerance | Overexpression of wheat phosphoenolpyruvate carboxylase kinase-related kinase gene (TaPEPKR2) | (Zang et al., 2018) |
| 1, 2, 13, 15 | increased tolerance of abiotic stress | RSR | wheat | increased drought tolerance | Overexpression of wheat STRESS ASSOCIATED PROTEIN 5 (TaSAP5) an E3 ubiquitin ligase that leads to the degradation of DRIP proteins that degrade DREB2A Transcription Factor | (Zhang et al., 2017a) |
| 1, 2, 13, 15 | increased tolerance of abiotic stress | RSR | wheat | increased tolerance to heat and drought | sunflower homeodomain-leucine zipper transcription factor (HB4) under the control of the maize ubiquitin promoter (ZmUBI1p:HaHB4) | (González et al., 2019) |
| 1, 2 | increased yield | RSR | canola | increased yield (biomass and seed weight) | quadruple KO of Clavata 3 | (Yang et al., 2018) |
| 1, 2 | increased yield | RSR | canola | increased yield, branching and seed weight), semi dwarf. | quadruple KO of MAX1 (cytochrome P450 monoxygenase involved in strigolactone synthesis) | (Zheng et al., 2020) |
| 1, 2, 13,15 | increased yield | RSR | canola | increased seed oil content | KO of SEED FATTY ACID REDUCER (SFAR4 or SFAR5) | (Karunarathna et al., 2020) |
| 1, 2, 15 | Increased yield | RSR | soybean | Increased yield, increased photosynthetic efficiency under fluctuating light | Overexpression of *A. thaliana* *ZEP* (Zeaxanthin epoxidase) using *AtRbcS* (RuBisCO small subunit 1A) promoter; *AtPsbS* (Photosystem II subunit S) using *AtGAPA-1* (Glyceraldehyde 3-phosphate dehydrogenase A subunit 1) promoter, and *AtVDE* (violaxanthin de-epoxidase) using *AtFBA2* (Fructose-bisphosphate aldolase 2 ) promoter, to accelerate the relaxation of nonphotochemical quenching during sun-shade transitions. | (De Souza et al, 2022) |
| 1,2,15 | increased yield | RSR | tef | Reduced lodging | Quadruple KO of gibberellin 20-oxidase 2 (*EtSD-1*), an enzyme in the biosynthesis of gibberellin. Naturally occurring mutations in rice (*sd-1*) were central in the green revolution. | (Beyene et al., 2022) |
| 1, 2, 15 | increased yield | RSR | tobacco | increased yield through reduced photorespiration | Expression of chloroplast targeted *Cucurbita maxima* malate synthase (CmMS), *Chlamydomonas reinhardtii* glycolate dehydrogenase CrGDH), RNA interference construct to reduce plastid glycolate-glycerate transporter PLGG1 | (Cavanagh et al., 2022) |
| 1, 2, 13,15 | increased yield and increased tolerance to abiotic stress | RSR | rice | increased yield, tolerance to salinity | Overexpression of ARGONAUTE2 (OsAGO2) (leading to activation of BIG GRAIN3 (OsBG3) (Purine Permease and putative cytokinin transporter) | (Yin et al., 2020) |
| 1, 2, 13,15 | increased yield and increased tolerance to abiotic stress | RSR | rice | increased yield, increased drought and salinity tolerance | Overexpression of RICE ABSCISSIC ACID STRESS AND RIPENINIG INDUCED (OsASR1) | (Park et al., 2020) |
| 1, 2, 13, 15 | increased yield and increased tolerance to abiotic stress | RSR | rice | increased yield, increased tolerance to drought, salinity, pH | ABA inducible expression of E. coli trehalose-6-phosphate synthase-trehalose-6-phosphate phosphatase fusion protein (EcTPSP) | (Joshi et al., 2019) |
| 1, 2, 13, 15 | increased yield and increased tolerance to abiotic stress | RSR | rice | increased yield, increased tolerance to drought, salinity, pH | stress induced expression OsNAC6 promoter fused to rice tandem zinc finger 5 (OsTZF5) | (Selvaraj et al., 2020) |
| 1, 2, 13,15 | increased yield and increased tolerance to abiotic stress | RSR | rice | increased yield, increased drought tolerance | Overexpression of Arabidopsis galactinol synthase 2 gene (AtGolS2) | (Selvaraj et al., 2017) |
| 1, 2, 13, 15 | increased yield and increased tolerance to abiotic stress | RSR | rice, potato | increased yield, increased drought tolerance, increased photosynthetic efficiency | expression of human RNA demethylase (HsFTO) | (Yu et al., 2021) |
| 1, 2, 14,15 | increased yield and reduced fertilizer requirement | RSR | rice | increased yield, increased nitrogen use efficiency | Co-overexpression of rice nitrate transporter (NRT2.3a) and partner protein (NAR2.1 a) from CaMV 35 S promoter | (Chen et al., 2020) |
| 1, 2, 3, 15 | increased yield, improved quality | RSR | rice | increased yield, increased fragrance (2-acetyl-1-pyrroline) | homozygous KO of betaine aldehyde dehydrogenase 2 (BADH2) (fragrance), homozygous KO and three P450 genes, Os03g03100, Os03g0568400, GL3.2 | (Usman et al., 2020b) |
| 1,2,13, 15 | increased yield, increased tolerance to abiotic stress | RSR | corn | increased yield and thermo tolerance | Overexpression of Arabidopsis glutaredoxin S17 (AtGRXS17) from maize ubiquitin-1 (Ubi-1) promoter | (Sprague et al., 2022) |
| 1, 2, 13,15 | increased yield, increased tolerance to abiotic stress | RSR | corn | increased yield and tolerance to drought | Overexpression of rice trehalose-6-phosphate phosphatase in corn ears using a rice floral promoter (OsMads6-Tpp1) | (Nuccio et al., 2015) |
| 1, 15 | increased yield, increased tolerance to abiotic stress | RSR | cotton | improved drought and heat tolerance, increased fiber yields | Overexpression of rice SUMO E3 Ligase (OsSIZ1) | (Mishra et al., 2017) |
| 1, 15 | increased yield, increased tolerance to abiotic stress | RSR | cotton | increased drought, heat, and salinity tolerance, increased fiber yields | Overexpression of rice SUMO E3 Ligase (OsSIZ1) and Arabidopsis vacuolar pyrophosphatase (AVP1) | (Esmaeili et al., 2021) |
| 1, 2, 15 | increased yield, increased tolerance to abiotic stress | RSR | rice | improved drought and heat tolerance, increased grain yield | Overexpression of rice Rab7 | (El-Esawi and Alayafi, 2019) |
| 1, 2, 13,15 | increased yield, increased tolerance to abiotic stress | RSR | wheat | increased crop water productivity (grain yield per liter of consumed water) | Overexpression of wheat ABA receptor 4 (TaPYL4) from the corn ubiquitin promter. | (Mega et al., 2019) |
| 1, 2, 13,15 | increased yield, increased tolerance to abiotic stress | RSR | wheat | increased yield under both well- watered and drought conditions, cytokinin mediated delay in senescence | Agrobacterium tumefaciens isopentenyl transferase under the control of a stress inducible promoter from Phaseolus vulgaris senescence associated receptor like kinase (PvSARKp:AtIPT). | (Beznec et al., 2021) |
| 1,2,6, 13,14, 15 | increased yield, increased tolerance to abiotic stress, reduced fertilizer requirement | RSR | wheat | increased yield under both drought and low-nitrogen | Overexpression of soybean TDN1, a DREB (dehydration responsive element binding protein)-like transcription factor from ubiquitin-1 (Ubi-1) promoter | (Zhou et al., 2022a) |
| 1, 2, 15 | reduced fertilizer requirement | RSR | rice | increased tolerance of potassium deficiency | Overexpression of Peroxiredoxin OsPrx2 (thiol-based peroxidase | (Mao et al., 2018) |
| 1, 2, 6, 13, 14,15 | reduced fertilizer requirement | RSR | tomato | increased tolerance to phosphate deficiency through increased phosphate uptake | expression of choline oxidase from *Arthrobacter globiformis* (AgcodA) | (Li et al., 2019) |
| 1, 2, 6, 13, 14,15 | reduced fertilizer requirement | RSR | rice | increased tolerance to low nitrogen | expression of cucumber alanine aminotransferase under the control of OsAnt1, a root specific promoter. | (Sisharmini et al., 2019) |

1. Could be exempt or could require an RSR [↑](#footnote-ref-1)
